# Atmospheric Pollutants Affect Physical Performance: A Natural Experiment in Horse Racing Studied by Principal Component Analysis

**DOI:** 10.3390/biology11050687

**Published:** 2022-04-30

**Authors:** Oscar F. Araneda, Gabriel Cavada

**Affiliations:** 1 Integrative Laboratory of Biomechanics and Physiology of Effort, LIBFE, Faculty of Medicine, School of Kinesiology, Universidad de los Andes, Santiago 8320000, Chile; 2 Faculty of Medicine, School of Public Health, University of Chile, Santiago 8320000, Chile; gcavada@med.uchile.cl

**Keywords:** air pollution, physical performance, thoroughbred racehorses

## Abstract

**Simple Summary:**

Thoroughbred horse racing is a natural experiment to study the effect of air pollutants on animal performance. In this activity, the animals are exposed to multiple mixtures of pollutants in the air and varying conditions of humidity and ambient temperature. Thus, in this work, in a homogeneous group of races, we used principal component analysis, which allowed us to gather information from all the environmental parameters measured, forming new variables called principal components. We found that the principal component is mainly determined as nitrogen oxides and carbon monoxide, while secondarily as particulate matter and sulfur oxides. Furthermore, this component is negatively related to the speed of the analyzed races. Thus, it is shown that air pollutants affect animal performance.

**Abstract:**

The impact of some atmospheric pollutants (PM_10_, PM_2.5_, O_3_, NO_2_, NO, SO_2_, CO), humidity and temperature were studied on the performance of thoroughbred racehorses. The study included 162 official handicap races held in 2012 in Santiago de Chile, at distances of 1000, 1100 and 1200 m, on a track in good condition, with a layout that included a bend, during the summer and winter months. The environmental variables were measured at the time of the race and were obtained from a monitoring station located 470 m from the equestrian center. The environmental variables showed an autocorrelation of variables, so they were reduced using principal component analysis. Subsequently, the principal components were correlated with running speed using Pearson’s method. Totals of 60.17 and 23.29% of the total variability of the data was explained by principal components 1 and 2 (PC1 and PC2), respectively. PC1 was mainly determined by NO, NO_2_, and CO (loadings~0.90) and secondarily by PM_10_, PM_2.5_, and SO_2_ (loadings~0.6), with which the data showed inverse associations, while with temperature and O_3_ it showed direct associations (loadings~0.7). In addition, this component correlated negatively with running speed (r = −0.50), while PC2 was not associated with this variable. In conclusion, using the principal component analysis strategy, it was determined that running speed is affected by air pollutants.

## 1. Introduction

Exercise implies an increase in metabolism and the appearance of more significant oxygen needs. Thus, it is necessary to increase airflow from the environment to capture this gas, resulting in bronchodilation, and increased tidal volume and respiratory rate. Once the O_2_ reaches the alveolar territory, it is incorporated into the bloodstream and circulates with a higher flow rate thanks to an increased systolic volume and heart rate. This gas will then be transported mainly into the erythrocytes to the skeletal muscle tissue, driven by the high-tissue-metabolic demand in the face of increased mechanical activity [1]. Horses are particularly advantageous for exercise, especially for athletes. This species has structural and functional advantages, including a low percentage of fat and an atypically high percentage of muscle mass, close to 55% of its weight [2].

Furthermore, in maximal exercise, it has been described that about 80% of cardiac output can perfuse skeletal muscle tissue [3]. Thus, among other factors, these factors explain why these animals have one of the highest peak-oxygen-consumption rates measured in different species (180–200 mL/kg/min) [4]. This high capacity to perform physical tasks has allowed the horse to participate in various tasks associated with activity such as agricultural work, transport, and military, activity after it was domesticated around 4000 BC [5]. Closely associated with this are the various equine sporting activities [6]. Thus, since the beginning of sports in which the participation of horses is required, these animals have been selected, bred, and trained to be athletes, with physical performance as a central aspect. This phenomenon has its maximum expression in the equestrian activities performed by thoroughbred racehorses from the seventeenth century [7]. From that time to the present day, horseracing has spread throughout the world, becoming a highly organized and specialized activity that brings together thousands of owners, breeders, trainers, and human and animal athletes competing in the most diverse environmental settings in temperature, humidity, and altitude. Another adverse environment for exercise is high levels of ambient air pollutants. There, associated with increased pulmonary ventilation, more pollutants will reach the organism since the air is a mixture in which there may be multiple unwanted components; among these, due to their frequency of occurrence and known biological effect, particulate matter (PM_10_ and PM_2.5_), nitrogen oxides (NO_2_ and NO), sulfur dioxide (SO_2_), carbon monoxide (CO) and ozone (O_3_), are usually present in varying concentrations in the air depending on the time of day, season, and geographical location [8].

Previously, many works have addressed the harmful effects of air pollutants, mainly concentrated on human health [9,10]; however, few works have been devoted to studying the effects of pollutants during exercise [11,12] and particularly on physical performance [13,14]. In this aspect, what is known has been developed in controlled studies where a pollutant has been applied under laboratory conditions [15,16]. Thus, it has been described that some pollutants affect lung function via bronchoconstriction and inflammation of the respiratory mucosa (particulate matter, NO_2_, O_3_, and SO_2_), alter the transport capacity of blood O_2_ (CO), and increase vasoconstrictor activity with cardiac alteration due to increased sympathetic activity (particulate matter, NO_2_, O_3_). Given the above, horse racing is a natural experiment where the problem of the relationship between air pollutants and performance can be studied. Despite the substantial body of evidence regarding organ effects that may eventually affect physical performance, the relationship between air pollutants and performance becomes complex to study since we breathe an air mixture that is highly changeable in composition and usually has more than one pollutant that affects performance [17]. If we think of an ideal design to study this relationship, the exercise model should be performed several times by the same pool of subjects who are similar in terms of their gene pool, training, nutrition, and basal-environmental exposure, set against multiple events that ensure a large variability air composition. Thus, to test this model, we generated a database of environmental variables composed of pollutant concentration, humidity, and temperature, in addition to the performance of horses in official races in the same equestrian center in the same season, to generate a model that relates both variables using principal component analysis.

## 2. Materials and Methods

*Sample*: The schedule, type of race, distance, sex, name, and time of the winning animals were obtained from the information published by the “Club Hípico de Santiago” (http://www.clubhipico.cl/, accessed on 1 February 2022), which corresponds to an open equestrian center located in the central sector of the city of Santiago, Chile. PM_10_ and PM_2.5_ concentrations were determined using thJane gravimetric method. O_3_ and nitrogen oxides (NO_2_ and NO) were measured using a chemiluminescent detector. CO was measured using dispersive infrared photometry, and sulfur oxides were measured using ultraviolet fluorescence. Finally, ambient temperature and humidity were measured using a thermometer and hygrometer, respectively. The consolidated data of these variables are published in the city’s air quality monitoring network (https://sinca.mma.gob.cl/, accessed on 1 February 2022) and were obtained from the “Parque O’Higgins” monitoring station, located 470 m from the equestrian enclosure.

*Database*: A database was constructed that included distance, type of race, track condition, time, average speed, name, age, and sex of the winning horse. The sample included 441 races held during the summer (January, February) and winter (May, June, July, and August) 2012. Of all races, those that reported a good track condition were selected, including up to one curve (1000, 1100 and 1200 m). Finally, regarding the type of race, according to our initial analysis [18], because it is a homogeneous group, the handicap-type races were selected, in which the participants, without restriction by sex, can gain access according to their previous performance and run in groups of similar abilities, and the chances of winning are balanced by the addition of weights according to their ranking. Regarding the data on environmental parameters, the values obtained at the time of the race were included in our analysis. Thus, our final sample corresponded to 162 races with all the environmental parameters (pollutants, humidity, and temperature) required by the principal component analysis method. In each race, approximately ten animals competed per test, which means that about 1600 animals participated. Our study only included the time of the winning animals, which corresponded to 64 females and 98 males of the English Thoroughbred breed. The animals were 5.00 (3.00–11.00) years old, expressed as median and range.

*Statistical analysis*: Initially, the distribution of the variables was determined utilizing the D’Agostino–Pearson test and the graphical analysis of their distribution. Then, the eventual differences in the animal’s speed according to their sex (Student’s-*t* test), the distances (1000, 1100, and 1200), and the month in which the races were carried out, were evaluated using the ANOVA test and Tukey’s test as a posteriori test. Similarly, the pollutant load was calculated according to the months, using ANOVA/Tukey or Kruskal-Wallis/Dunn’s, according to data distribution. Before performing the principal component analysis, the log function was applied to the non-normally distributed variables to reduce the effect of strongly skewed parameters in the principal component calculations. Subsequently, the measured environmental parameters were correlated. Thus, a high autocorrelation between them was determined. The principal components were then calculated, and those that explained the greatest variability in the data (a priori over 80%) were chosen for the subsequent study. Then, the correlations between the environmental parameters and the selected principal components were determined, taking an R greater than 0.60 as a relevant value. Finally, Pearson’s correlation coefficient was determined between running speed and the principal components considered. The significance level used in the tests corresponded to 5%. Statistical analysis was performed using GraphPad Prism (version 9.0; San Diego, CA, USA).

## 3. Results

**Figure 1:**
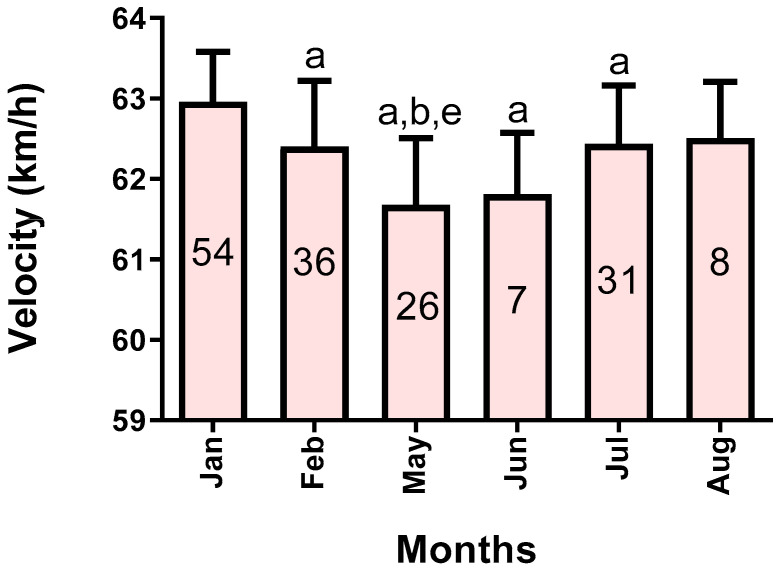
Running speed values grouped by months. The number of runs by group is presented in the middle part of each bar. Data are presented as mean and standard deviation. a = different from January, b = different from February, e = different from July, with a *p*-value < 0.05.

Using the D’Agostino–Pearson test, we found that PM_10_, O_3_, CO, NO_2_, NO, and SO_2_ showed a distribution different from normal, so the logarithmic function was applied prior to the calculation of the principal components. PM_2.5_, humidity, and temperature showed a normal distribution and were included with their original values. Regarding the analysis according to sex, the males had an average speed of 62.26 ± 0.95, while the females showed a speed of 62.36 ± 0.91 km/h, with no differences (*p* = 0.41). There were also no differences between the speeds displayed at the distances of 1000 (62.11 ± 0.95), 1100 (62.31 ± 0.99), and 1200 m (62.41 ± 0.55), with a value of *p* = 0.37. These results allowed us to gather the environmental data without differentiating by animal’s sex or the race´s distance. The speeds according to the months (see Figure 1) in which the tests were performed were different (*p* < 0.0001). Thus, we found a higher speed in the races carried out in January 62.96 ± 0.62, than February (62.40 ± 0.81; *p* < 0.01), May (61.68 ± 0.82; *p* < 0.0001), June (61.81 ± 0.76; *p* < 0.01), and July (62.43 ± 0.72; *p* < 0.05), while the speeds of the May races were lower than those of February (*p* < 0.01) and July (*p* < 0.01).

**Figure 2:**
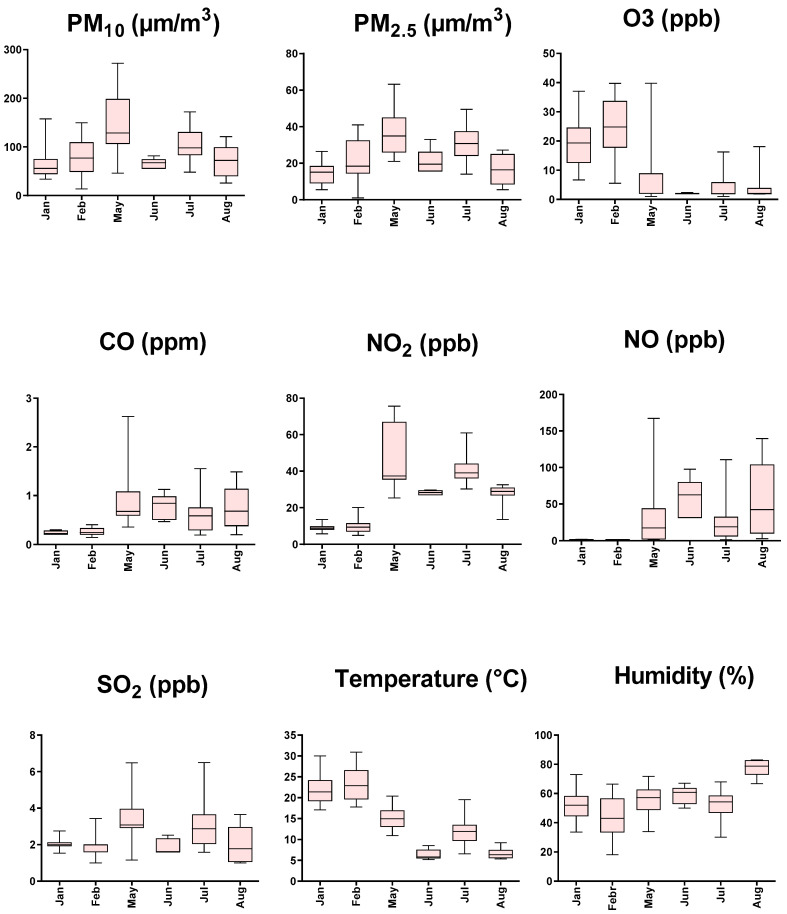
Air pollutant concentration, temperature, and humidity at the moment of races. Data are presented as box plots. The box consists of the first quartile, median and third quartile values, while the upper and lower horizontal lines are the minimum and maximum values, respectively. Statistical analysis is presented in Tables S2 and S3 of the Supplementary Material.

**Figure 3:**
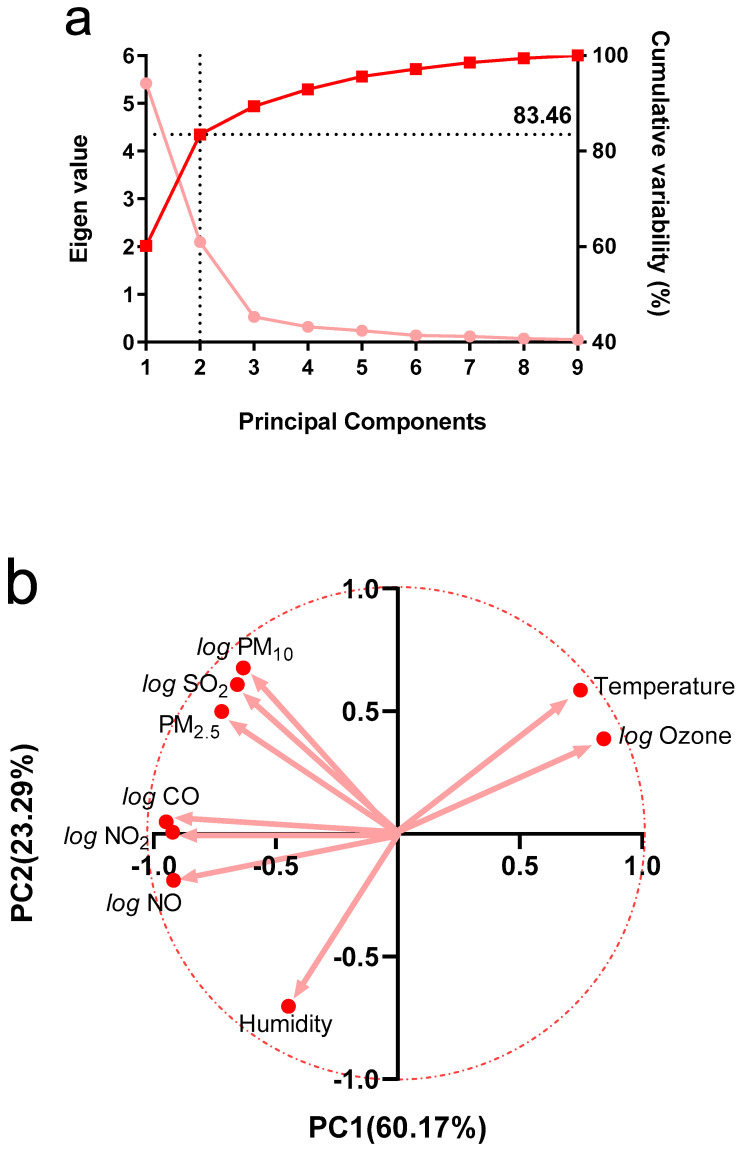
Eigenvalues and cumulative variability versus principal components (**a**). Biplot of PC1 versus PC2 (**b**). The arrow size represents the contribution of each variable, while location represents the sign of the association with each component.

**Figure 4:**
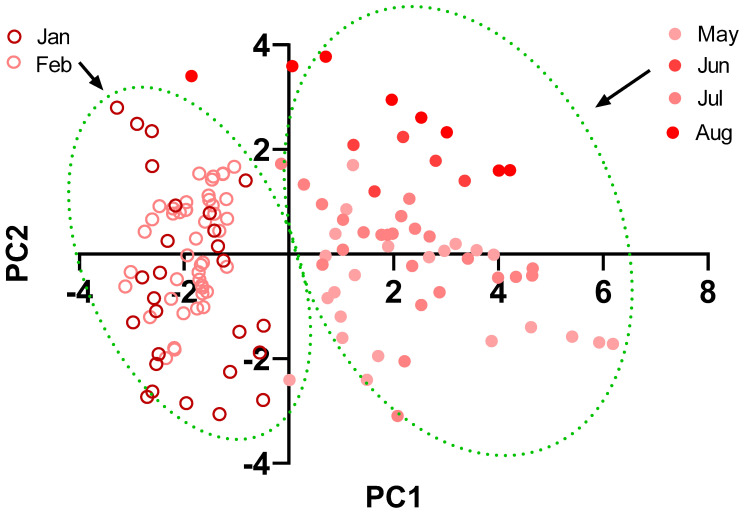
Individual values of PC1 versus PC2. The filled circles represent the values from the winter months, while the empty circles represent the values from the summer months.

**Figure 5:**
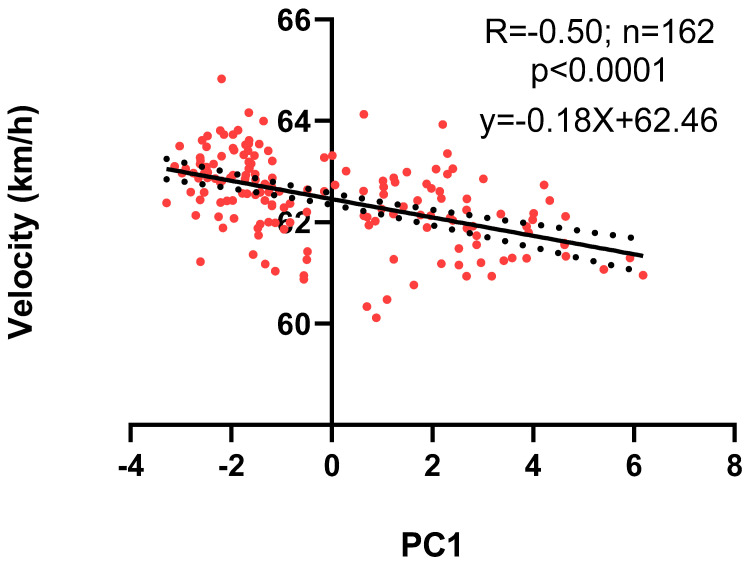
Running speed versus PC1. The trend line (filled) and the 95% confidence interval (dotted) are presented in black.

Pollutant´s concentration, humidity, and environmental temperature to which the animals were exposed at the time of the run are observed in Table S1 of the Supplementary Material. Figure 2 shows graphs of the same variables separated by months. We can see a tendency of an increase in the values of pollutants from January to May in PM_10_, PM_2.5_, CO, NO_2_, NO, and SO_2_. Then, a decrease is observed in June, rising in July, and falling again in August for PM_10_, P_2.5_, NO_2_, SO_2_, and temperature, while CO and NO decrease from June to July to rise again in August. Regarding O_3_, it shows similar levels in January and February, with lower and stable values for the rest of the months. Finally, humidity shows relatively stable values with a rise in August (see statistical analysis in Tables S2 and S3 of the Supplementary Material). The analysis of the correlation between the variables, in raw values or after applying the logarithm, shows a significant degree of autocorrelation, as can be seen in Table 1. The principal components analysis showed that components 1 and 2 explain 60.17 and 23.29% of the sample variance, respectively (Figure 3a). PC1 is strongly determined by NO_2_, NO, and CO (loadings above 0.9), while secondarily by PM_10_, PM_2.5_, and SO_2_ (loadings above 0.6). Furthermore, these pollutants were negatively associated with this component. PC1 is also positively associated with O_3_ and temperature (loadings above 0.7), as shown in Figure 3b and Table S4 of the Supplementary Material. PC2 is mainly determined by humidity (loading 0.70), and negatively associated with PM_10_ and SO_2,_ which is positively associated with loadings of 0.68 and 0.61, respectively. Figure 4 shows the relationship between PC1 versus PC2 values. It is observed that the summer months (January and February) have negative values for PC1, while the winter months (May, June, July, and August) have positive values. Regarding the correlations, speed was not significantly associated with PC2 (r = 0.08, *p* = 0.27), while, with PC1, it showed an inverse association (r = −0.50, *p* = 0.0001), as shown in Figure 5.

## 4. Discussion

Our data show that selected environmental variables affect the performance of highly trained animal athletes. For its realization, we took advantage of a natural experiment in which a relatively homogeneous group of animals of the same breed, age, similar environmental exposure, training conditions, and diet participate in equestrian competitions. To cross-check performance with air pollutants, we selected the summer and winter months to favor the maximum variability in environmental conditions (see Figure 2) and generate a model of the relationship between these variables. We tried to reduce the factors that may affect the relationship under study regarding performance. Thus, we previously found that the times of winning horses in handicap-type races with a track in good condition, in speed trials (<1200 m), correlate, mostly negatively, with the concentration of air pollutants, constituting an attractive experimental model [16]. Since the environmental variables move in a block, which we observed by their autocorrelation (see Table 1), we proceeded to employ the methodology of principal component analysis to reduce the variables to new ones without losing the information of the primary ones [19]. This experimental approach is relevant because most of the works that have studied this relationship were carried out with one or two pollutants [20,21], while, in natural conditions, we breathe a mixture of them.

Our results evidenced a decrease in the speed of the winning horses during the winter months (particularly in May/June), as shown in Figure 1. In the direction of this finding, pollutants, except O_3_, had an inverse pattern of variation in their concentrations, as shown in Figure 2. Likewise, we saw that, when plotting the relationship between the two principal components that explain most of the variability of the data (PC1 and PC2), only PC1 can discriminate between summer versus winter environmental data, which corresponds to the time when the drop in animal performance was appreciated (see Figure 4). Concerning the determinants of PC1, it is possible to recognize three clusters of variables grouped. Firstly, those that most determined this component are nitrogen oxides (NO and NO_2_), known to generate pro-inflammatory effects and increase airway resistance [22], which could prevent the achievement of sufficient bronchodilation necessary to increase airflow during exercise. We found carbon monoxide to be very close in terms of the magnitude of influence and the direction of its relationship with PC1. The interesting thing about this phenomenon is that this gas would affect performance by very different mechanisms than nitrogen oxides, since it affects oxygen transport via the formation of carboxyhemoglobin [23] and can eventually affect the functioning of organs such as the heart [24]. Secondarily, PC1 is determined by another group of similarly associated pollutants, such as PM_10_, PM_2.5_, and SO_2_. These pollutants act at the pulmonary level by similar mechanisms, by being pro-inflammatory substances, irritants, and favoring bronchoconstriction [25,26]. Concerning O_3_, it was positively related to PC1. At first, this may seem paradoxical because of its known effects as an irritant and as a pro-inflammatory substance; however, the levels reported here (median 10.60 ppb) are lower than the concentrations described as triggering the effects of this gas in humans (100–600 ppb) [27]. In addition, it is relevant to note that our group of animals is accustomed to exercising in poor air conditions, which may reduce the response to this gas [28] and other pollutants. Another important aspect is the inclusion of humidity and temperature in our model, both because they influenced the concentration of pollutants and their possible effect on performance. We found that humidity significantly affects PC2 but does not affect PC1. Thus, according to our findings, it has a poor role in performance. Temperature possesses a favorable effect on animal performance between the 5 and 30 °C presented in our sample [29]. The above confirms the direction of our results, since this variable is positively related to PC1. In addition, it stands out that it is presented very close in the biplot graph to O_3_, due to the need for photochemical reactions for its formation, which in part can explain the relationship between this pollutant and PC1.

Once we knew the contribution of each pollutant on different principal components, we proceeded to study the relationship between these with physical performance. For this, we correlated running speed with PC1 and PC2, respectively. Thus, as mentioned, only PC1 showed an inverse association with the velocity of winning horses, which shows the deleterious effect of pollutants on performance. This result is relevant because it is related to biological effects described for pollutants, as well as with the results found by previous studies in this regard [30,31,32].

Two aspects are of interest concerning the relationship found: Firstly, to determine the importance of humidity and temperature in our results. To resolve this question, we extracted both variables from the model, finding that the relationship velocity versus PC1 shows a value of r = −0.51. It follows that pollutants are apparently the main variables explaining the relationship encountered. Another interesting aspect is the temporality of exposure and its effect on the relationship studied. We performed the same analysis using the pollutant concentrations six hours before the races (see the analysis in Supplementary Material on Tables S5–S11 and Figures S1–S3). Thus, we found that the relationship is maintained, but the strength of the association decreases (r = −0.34). A likely explanation may be that the immediate effects of pollutants predominate over the slow ones. Still, this idea should be taken with caution because of the correlations between the six hours before values versus the immediate (see Supplementary Material Table S9).

A limitation of the study is that we have not performed the determinations immediately at the edge of the track or in sampling devices installed on the animals, which could have an influence, particularly considering that some races were run on a sand track, which could generate an increase in particulate matter. In addition, we do not have measurements of pre-race exposure to pollutants inherent to the activity, such as in the animal pens.

The reported findings are important to all those involved in equestrian activity, as this may affect training schedules, programming, or even deciding on the participation of animals in adverse environmental conditions that are not currently considered for racing. In addition, they provide relevant information for exercise physiology and training in humans, due to the ethical and technical difficulties in developing a protocol that includes so many high-performance athletes exposed to such diverse ambient air conditions. Finally, our findings should be confirmed in a larger number of races and over longer distances in the future.

## 5. Conclusions

Based on field data from an equestrian center, we found that physical performance is affected by pollutants in the ambient air; in particular, this phenomenon is associated with several pollutants, including nitrogen oxides, CO, particulate matter, and SO_2_.

## Figures and Tables

**Table 1:** Pearson correlation coefficients for temperature, humidity, and pollutant concentration. The values in bold type have a *p*-value lower than 0.05.

	log PM_10_	PM_2.5_	log O_3_	log CO	log NO_2_	log NO	log SO_2_	Temperature	Humidity
log PM_10_	**1.00**								
PM_2.5_	**0.78**	**1.00**							
log O_3_	**−0.26**	**−0.34**	**1.00**						
log CO	**0.62**	**0.70**	**−0.74**	**1.00**					
log NO_2_	**0.55**	**0.67**	**−0.79**	**0.82**	**1.00**				
log NO	**0.47**	**0.49**	**−0.88**	**0.87**	**0.79**	**1.00**			
log SO_2_	**0.74**	**0.67**	**−0.33**	**0.66**	**0.59**	**0.51**	**1.00**		
Temperature	−0.08	**−0.25**	**0.84**	**−0.65**	**−0.72**	**−0.78**	−0.12	**1.00**	
Humidity	**−0.14**	0.09	**−0.53**	**0.43**	**0.34**	**0.45**	−0.12	**−0.66**	**1.00**

## Data Availability

The database is included in the supplementary material.

## Supplementary Materials

The following supporting information can be downloaded at: https://www.mdpi.com/article/10.3390/biology11050687/s1, Analysis with environmental data collected at the time of the races. Table S1: Temperature, humidity, and concentration of pollutants. Table S2: Mean and standard deviation of pollutant, temperature, and humidity by months. Table S3: p-values from pairwise comparisons in pollutants, humidity, and temperature between months. Table S4: Correlation coefficients between the absolute values or their logarithm versus the components PC1 and PC2. Analysis with environmental data collected six hours before the races. Table S5: Temperature, humidity, and concentration of pollutants. Table S6: Mean and standard deviation of pollutant, temperature, and humidity by months. Table S7: *p*-values from pairwise comparisons in pollutants, humidity, and temperature between months. Table S8: Correlation coefficients between absolute values or their logarithm versus PC1 and PC2 components. Table S9: Pearson’s correlation coefficients between the absolute values of pollutants, humidity and temperature obtained at the time of the races versus six hours previous values. Table S10: Pearson correlation coefficients for temperature, humidity, and air pollutant concentration. Table S11: Database of handicap races. Figure S1: Air pollutant concentration, temperature, and humidity. Figure S2: Eigen values and cumulative variability versus principal components and biplot of PC1 versus PC2. Figure S3: PC1 versus running speed relationship.
