# Supplementary material for: Atmospheric Pollutants Affect Physical Performance: A Natural Experiment in Horse Racing Studied by Principal Component Analysis
Source: Biology (Basel). 2022 Apr 30;11(5):687. doi: 10.3390/biology11050687 (PMC9138079; doi:10.3390/biology11050687)
Supplement: Supplementary file 1 [file biology-11-00687-s001.zip › biology-1666268-supplementary.pdf]

Supplementary Materials

a) Analysis with environmental data (pollutants, humidity, temperature) collected at the time of the races.

Table S1. Temperature, humidity, and concentration of pollutants measured in the air at the time of the races.

	PM_{10}	$PM_{2.5}$	O_3	CO	NO_2	NO	SO_2	Temperature	Humidity
Unit	mg/m³	mg/m³	ppb	ppm	ppb	ppb	ppb	°C	%
Minimum	13.50	1.00	1.00	0.10	4.90	1.00	1.00	5.20	18.10
25% Percentile	50.80	14.70	2.10	0.20	8.80	1.00	1.90	12.90	44.60
Median	78.90	20.00	10.60	0.30	13.10	1.20	2.00	18.70	53.30
75% Percentile	113.30	31.00	22.10	0.60	36.10	19.10	2.90	22.40	60.80
Maximum	272.0	63.30	39.80	2.60	75.60	167.30	6.50	30.90	82.90
Mean	88.90	22.90	14.10	0.50	23.00	16.40	2.50	17.80	52.40
Std. Deviation	48.10	12.20	11.70	0.40	17.50	31.10	1.00	6.40	12.50

Table S2. Mean and standard deviation of pollutant, temperature, and humidity at the time of the races by months. The parameters that showed a normal distribution are shown in bold.

**Month**	**n**	**PM_{10} (mg/m³)**	**$PM_{2.5}$ (mg/m³)**	**O_3 (ppb)**	**CO (ppm)**	**NO_2 (ppb)**	**NO (ppb)**	**SO_2 (ppb)**	**Temperature (°C)**	**Humidity (%)**
**January**	54	55.75(44.19-75.00)	14.46±5.44	19.34(12.52-24.59)	0.22(0.20-0.28)	8.83(7.94-9.87)	1.00(1.00-1.03)	2.00(1.91-2.12)	21.82±3.35	51.91±10.1
**February**	36	77.00(48.63-109.50)	21.36±11.78	24.83(17.73-33.72)	0.24(0.20-0.33)	9.46(6.99-11.41)	1.00(1.00-1.00)	2.00(1.59-2.00)	23.19±3.81	43.48±13.68
**May**	26	128.60(106.40-198.70)	36.20±12.30	2.10(1.99-8.94)	0.67(0.58-1.08)	37.37(35.36-67.05)	17.55(1.83-44.06)	3.08(2.91-3.96)	15.14±2.46	56.30±9.12
**Juny**	7	67.50(55.00-74.50)	20.68±6.66	1.99(1.99-2.20)	0.84(0.50-0.98)	28.33(26.83-29.50)	62.58(30.92-80.08)	1.59(1.59-2.34)	6.54±1.30	58.31±6.44
**July**	31	98.00(83.00-130.50)	30.54±9.03	1.98(1.90-5.87)	0.58(0.29-0.75)	39.00(36.04-44.09)	19.12(5.84-32.38)	2.87(2.03-3.66)	11.94±2.99	52.69±8.76
**August**	8	72.38(39.81-99.38)	16.34±8.35	1.98(1.98-3.94)	0.67(0.37-1.13)	28.92(26.68-30.94)	42.24(9.90-104.10)	1.78(1.05-2.96)	6.62±1.32	77.37±5.90

Table S3. P-values from pairwise comparisons in pollutants, humidity, and temperature at the time of the races between months. The ANOVA test followed by Tukey's test was applied to the columns in bold, while the Kruskal Wallis test and Dunn's test were applied to the unmarked columns. *p<0.05, **p<0.01, ***p<0.0001, ****p<0.00001.

	**PM_{10}**	**$PM_{2.5}$**	**O_3**	**CO**	**NO_2**	**NO**	**SO_2**	**Temperature**	**Humidity**
	(mg/m³)	**(mg/m³)**	(ppb)	(ppm)	(ppb)	(ppb)	(ppb)	**(°C)**	**(%)**
**January vs. February**	ns	*	ns	ns	ns	ns	ns	ns	**
**January vs. May**	****	****	****	****	****	****	****	****	ns
**January vs. Juny**	ns	ns	**	***	ns	****	ns	****	ns
**January vs. July**	***	****	****	****	****	****	***	****	ns
**January vs. August**	ns	ns	***	**	*	****	ns	****	****
**February vs. May**	****	***	****	****	****	****	****	****	****
**February vs. Juny**	ns	ns	***	**	ns	****	ns	****	**
**February vs. July**	ns	*	****	***	****	****	***	****	**
**February vs. August**	ns	ns	****	**	ns	****	ns	****	****
**May vs. Juny**	****	ns	ns	ns	ns	ns	**	****	ns
**May vs. July**	***	ns	ns	ns	ns	ns	ns	**	ns
**May vs. August**	****	**	ns	ns	ns	ns	**	****	****
**Juny vs. July**	ns	ns	ns	ns	ns	ns	ns	***	ns
**Juny vs. August**	ns	ns	ns	ns	ns	ns	ns	ns	**
**July vs. August**	ns	*	ns	ns	ns	ns	ns	***	****

Table S4. Correlation coefficients between the absolute values or their logarithm versus the components PC1 and PC2 at the time of the races.

Parameter	PC1	PC2
log PM_{10}	-0.63	0.68
$PM_{2.5}$	-0.72	0.50
log Ozone	0.84	0.39
log CO	-0.95	0.05
log NO_2	-0.92	0.01
log NO	-0.92	-0.18
log SO_2	-0.66	0.61
Temperature	0.75	0.59
Humidity	-0.45	-0.70

a) Analysis with environmental data (pollutants, humidity, temperature) collected six hours before the races.

Table S5. Temperature, humidity, and concentration of pollutants measured in the air six hours before the races.

	PM_{10}	$PM_{2.5}$	O_3	CO	NO_2	NO	SO_2	Temperature	Humidity
**Unit**	**mg/m³**	**mg/m³**	**ppb**	**ppm**	**ppb**	**ppb**	**ppb**	**°C**	**%**
**Minimum**	22.00	4.70	4.10	0.20	5.40	1.00	1.00	7.50	17.90
**25% Percentile**	64.30	15.50	14.60	0.20	6.90	1.00	2.00	18.10	33.40
**Median**	88.30	20.20	30.20	0.30	8.80	1.40	2.30	24.00	39.00
**75% Percentile**	108.70	28.80	36.80	0.50	38.90	16.40	3.00	27.40	45.50
**Maximum**	183.10	65.70	55.70	1.50	79.70	129.20	5.80	33.00	73.50
**Mean**	89.40	22.70	27.10	0.40	24.20	9.60	2.60	22.80	39.80
**Std. Deviation**	29.40	11.10	12.80	0.20	22.10	15.80	1.00	6.30	11.30

Table S6. Mean and standard deviation of pollutant, temperature, and humidity six hours before the races by months. The parameters that showed a normal distribution are shown in bold.

**Month**	**n**	**PM_{10} (mg/m³)**	**$PM_{2.5}$ (mg/m³)**	O_3 (ppb)	CO (ppm)	NO_2 (ppb)	NO (ppb)	SO_2 (ppb)	**Temperature (°C)**	**Humidity (%)**
**January**	54	79.63±19.59	16.62±4.87	33.48±5.98	0.2254±0.02	6.92±1.10	1.176±0.36	2.16±0.28	26.91±2.47	41.14±7.51
**February**	36	84.52±21.32	18.24±2.61	37.75±7.50	0.2403±0.04	7.72±1.26	1.226±0.50	2.14±0.58	27.91±3.03	34.35±13.00
**May**	26	113,00±31.67	32.33±8.03	27.5±12.12	0.5908±0.15	45.14±14.00	11.27±8.61	3.73±0.89	20.9±2.53	36.63±7.24
**Juny**	31	114,1±13.71	37.07±8.96	11.27±4.34	0.6467±0.30	55.62±12.99	29.02±24.51	3.36±1.07	17.37±1.67	37.12±4.80
**July**	7	54,99±2.90	10.77±2.05	8.813±1.88	0.3875±0.04	30.37±0.21	19.82±3.56	2.36±0.12	11.72±0.88	46.05±1.27
**August**	8	34.69±10.92	8.00±3.33	12.13±3.21	0.343±0.11	19.26±3.42	13.69±11.84	1.20±0.29	8.29±0.52	70.95±1.93

Table S7. P-values from pairwise comparisons in pollutants, humidity, and temperature six hours before the races between months. The ANOVA test followed by Tukey's test was applied to the columns in bold, while the Kruskal Wallis test and Dunn's test were applied to the unmarked columns. *p<0.05, **p<0.01, ***p<0.0001, ****p<0.00001.

	**PM_{10} (mg/m³)**	**$PM_{2.5}$ (mg/m³)**	**O_3** (ppb)	**CO** (ppm)	**NO_2** (ppb)	**NO** (ppb)	**SO_2** (ppb)	**Temperature (°C)**	**Humidity (%)**
**January vs. February**	ns	ns	ns	ns	ns	ns	ns	ns	**
**January vs. May**	****	****	ns	****	****	****	****	****	ns
**January vs. Juny**	****	****	****	****	****	****	****	****	ns
**January vs. July**	*	ns	***	*	**	***	ns	****	ns
**January vs. August**	****	**	**	ns	*	**	*	****	****
**February vs. May**	****	****	ns	****	****	****	****	****	ns
**February vs. Juny**	****	****	****	****	****	****	****	****	ns
**February vs. July**	**	*	****	ns	ns	****	ns	****	*
**February vs. August**	****	***	****	ns	ns	***	*	****	****
**May vs. Juny**	ns	*	****	ns	ns	ns	ns	****	ns
**May vs. July**	****	****	**	ns	ns	ns	ns	****	ns
**May vs. August**	****	****	*	ns	ns	ns	****	****	****
**Juny vs. July**	****	****	ns	ns	ns	ns	ns	****	ns
**Juny vs. August**	****	****	ns	ns	ns	ns	****	****	****
**July vs. August**	ns	ns	ns	ns	ns	ns	*	ns	****

Table S8. -Correlation coefficients between absolute values or their logarithm versus PC1 and PC2 components measured six hours before the races.

Parameter	PC1	PC2
**PM_{10}**	-0.61	0.63
**$logMP_{2.5}$**	-0.72	0.53
**Ozone**	0.65	0.64
***log* CO**	-0.93	0.05
***log* NO_2**	-0.97	-0.06
***log* NO**	-0.91	-0.29
***log* SO_2**	-0.66	0.61
**Temperature**	0.63	0.72
**Humidity**	-0.01	-0.83

Table S9. Pearson's correlation coefficients between the absolute values of pollutants, humidity and temperature obtained at the time of the races versus six hours previous values.

Parameter	Pearson´s Coefficient
**PM_{10}**	0.63
**$PM_{2.5}$**	0.63
**O3**	0.78
**CO**	0.63
**NO_2**	0.90
**NO**	0.32
**SO_2**	0.71
**Temperature**	0.92
**Humidity**	0.79

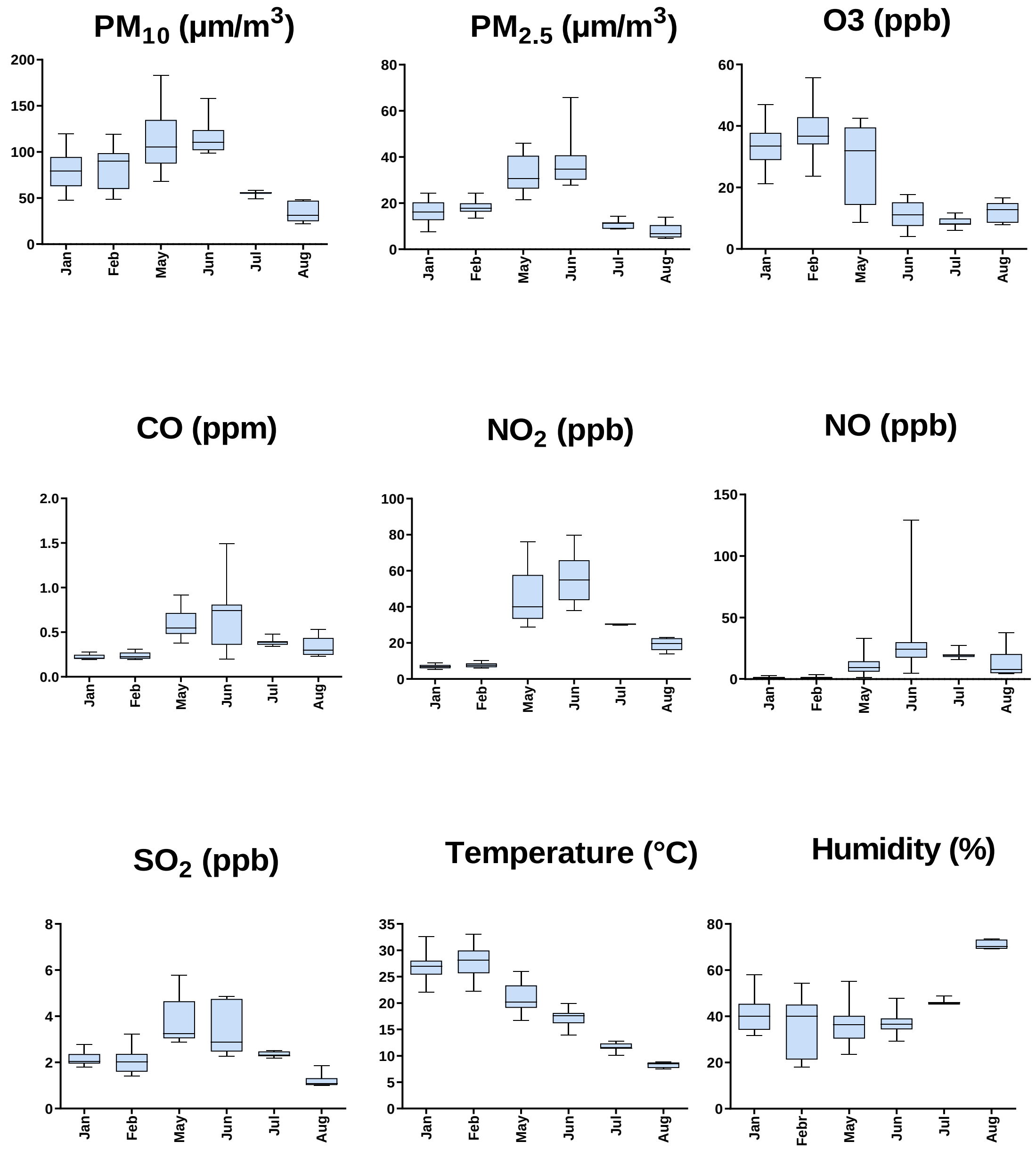

Figure S1. Air pollutant concentration, temperature, and humidity six hours before the races. Data are presented as box plots. The box consists of the first quartile, median and third quartile values, while the upper and lower horizontal lines are the minimum and maximum values respectively. Statistical analysis is presented in tables S6 and S7.

Table S10. Pearson correlation coefficients for temperature, humidity, and air pollutant concentration six hours before the races. The values represented in bold have a p-value of less than 0.05.

	PM_{10}	*log* $PM_{2.5}$	O_3	*log* CO	*log* NO_2	*log* NO	*log* SO_2	Temperature	Humidity
**PM_{10}**	**1.00**								
log **$PM_{2.5}$**	**0.78**	**1.00**							
**O_3**	*-0.02*	*-0.11*	**1.00**						
log **CO**	**0.58**	**0.68**	**-0.50**	**1.00**					
log **NO_2**	**0.50**	**0.64**	**-0.65**	**0.88**	**1.00**				
log **NO**	**0.30**	**0.46**	**-0.80**	**0.83**	**0.91**	**1.00**			
log **SO_2**	**0.70**	**0.71**	*-0.04*	**0.59**	**0.61**	**0.42**	**1.00**		
**Temperature**	*0.05*	*-0.05*	**0.84**	**-0.51**	**-0.68**	**-0.75**	*-0.02*	**1.00**	
**Humidity**	**-0.39**	**-0.29**	**-0.44**	*0.00*	*0.01*	**0.16**	**-0.50**	**-0.59**	**1.00**

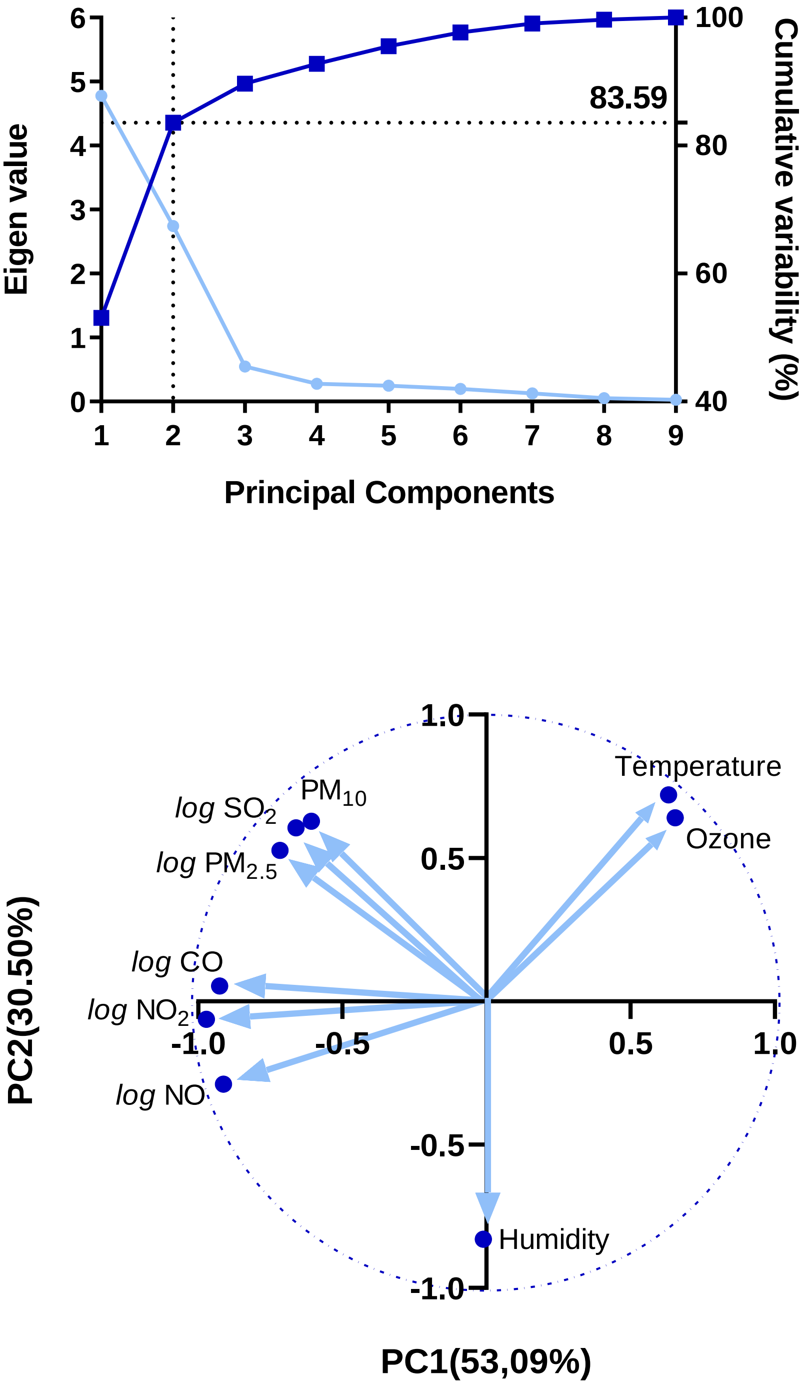

Figure S2. - Eigen values and cumulative variability versus principal components calculated six hours before the races (above). Biplot of PC1 versus PC2. The size of the arrows represents the magnitude of the variable's contribution, while their location represents the sign of the association with each component (bottom).

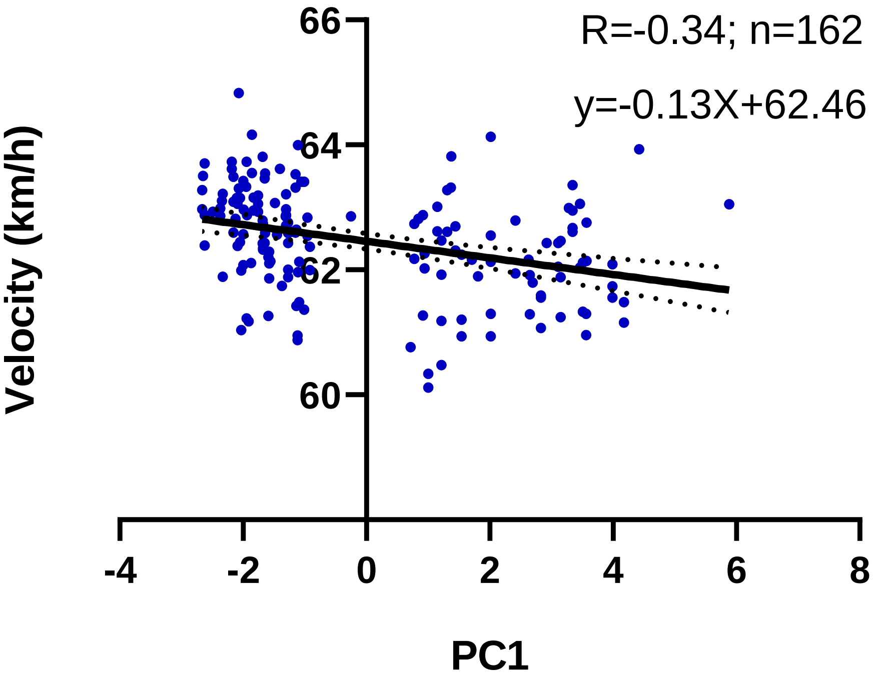

Figure S3. - PC1 versus running speed relationship. The trend line (filled) and the 95% confidence interval (dotted) are presented in black.

Table S11. Database of handicap races

Number	Sex	Month	Distance	Velocity	*PM10*	*PM2_5*	*Ozone*	*CO*	*NO2*	*NO*	*SO2*	*Temperature*	*Humidity*	**PM10**	**PM2_5**	**Ozone**	**CO**	**NO2**	**NO**	**SO2**	**Temperature**	**Humidity**
10	F	JAN	1100	63.15	81.75	19.00	35.46	0.20	8.38	1.00	2.76	30.01	34.86	74.67	22.25	45.40	0.20	6.27	1.00	2.62	32.45	33.13
11	F	JAN	1100	64.83	81.50	20.00	31.08	0.20	9.12	1.00	2.63	28.84	36.42	76.92	20.58	44.82	0.20	6.37	1.00	2.78	32.55	32.30
12	M	JAN	1200	63.31	81.50	20.00	31.08	0.20	9.12	1.00	2.63	28.84	36.42	76.92	20.58	44.82	0.20	6.37	1.00	2.78	32.55	32.30
14	F	JAN	1100	62.88	63.00	19.50	23.78	0.20	10.45	1.00	2.55	26.18	40.42	80.00	20.33	42.52	0.20	6.91	1.00	2.77	31.99	32.73
15	M	JAN	1100	63.06	65.50	17.00	18.94	0.23	13.52	1.00	2.47	24.22	43.92	79.25	20.25	38.64	0.20	7.78	1.00	2.74	30.86	34.18
16	F	JAN	1100	63.19	65.50	17.00	18.94	0.23	13.52	1.00	2.47	24.22	43.92	79.25	20.25	38.64	0.20	7.78	1.00	2.74	30.86	34.18
17	F	JAN	1100	62.93	56.50	16.00	18.77	0.24	12.82	1.00	2.43	23.57	45.00	79.25	20.25	38.64	0.20	7.78	1.00	2.74	30.86	34.18
24	M	JAN	1100	62.82	157.50	16.00	29.30	0.23	5.79	1.25	1.98	28.00	37.33	64.75	12.08	34.76	0.27	5.98	1.29	1.79	27.20	37.42
29	M	JAN	1100	63.33	140.50	15.50	18.51	0.21	6.28	1.83	1.98	24.40	45.08	95.42	11.75	32.58	0.25	5.37	1.08	1.91	28.04	36.05
30	M	JAN	1100	62.32	120.50	12.50	12.14	0.29	8.19	2.00	1.90	21.46	51.67	109.17	12.08	28.93	0.24	5.50	1.22	1.92	27.50	37.30
31	M	JAN	1100	62.35	120.50	12.50	12.14	0.29	8.19	2.00	1.90	21.46	51.67	109.17	12.08	28.93	0.24	5.50	1.22	1.92	27.50	37.30
32	F	JAN	1100	62.88	75.00	9.00	9.09	0.30	9.84	1.58	1.90	19.27	57.08	119.75	12.67	24.88	0.24	6.05	1.39	1.94	26.40	39.99
33	F	JAN	1100	63.21	75.00	9.00	9.09	0.30	9.84	1.58	1.90	19.27	57.08	119.75	12.67	24.88	0.24	6.05	1.39	1.94	26.40	39.99
34	M	JAN	1100	62.71	57.00	8.50	7.90	0.30	10.18	1.58	1.94	18.62	58.88	119.75	12.67	24.88	0.24	6.05	1.39	1.94	26.40	39.99
35	M	JAN	1100	63.41	39.00	8.00	6.71	0.30	10.51	1.58	1.98	17.96	60.67	119.50	12.42	21.23	0.25	6.89	1.48	1.94	24.85	43.92
47	F	JAN	1100	63.07	94.00	24.00	33.37	0.28	9.09	1.03	2.04	25.45	50.33	104.42	24.33	46.99	0.27	8.33	1.05	1.94	28.61	40.46
48	M	JAN	1100	63.61	82.00	23.00	26.49	0.26	8.85	1.00	1.70	22.75	57.00	104.17	24.08	43.61	0.27	8.14	1.03	1.97	28.09	42.31
50	F	JAN	1100	62.76	63.00	18.50	20.80	0.30	10.41	1.00	1.95	20.57	62.83	102.17	24.17	38.98	0.27	8.26	1.03	1.95	27.02	45.46
51	M	JAN	1100	62.43	63.00	18.50	20.80	0.30	10.41	1.00	1.95	20.57	62.83	102.17	24.17	38.98	0.27	8.26	1.03	1.95	27.02	45.46
52	M	JAN	1100	62.59	54.75	16.50	19.70	0.30	9.98	1.00	1.91	19.91	64.46	102.17	24.17	38.98	0.27	8.26	1.03	1.95	27.02	45.46
53	F	JAN	1100	63.32	46.50	14.50	18.60	0.29	9.54	1.00	1.87	19.25	66.08	94.58	23.17	34.75	0.28	8.74	1.02	1.94	25.57	49.56
54	M	JAN	1100	63.53	44.50	14.50	17.37	0.26	9.24	1.00	1.53	18.57	68.13	94.58	23.17	34.75	0.28	8.74	1.02	1.94	25.57	49.56
60	M	JAN	1200	62.87	69.25	16.75	32.23	0.20	5.87	1.07	2.19	28.04	38.75	66.08	16.33	27.27	0.23	7.89	2.99	1.98	24.69	47.45
65	M	JAN	1100	63.73	58.50	18.50	22.59	0.20	7.48	1.11	2.19	24.32	46.58	62.50	14.67	31.08	0.20	5.43	1.05	2.08	27.11	40.77
67	F	JAN	1100	62.08	55.00	16.50	18.26	0.21	8.94	1.01	2.11	22.39	50.17	62.92	16.00	30.15	0.20	5.76	1.05	2.19	26.94	41.04
68	M	JAN	1100	63.42	55.00	16.50	18.26	0.21	8.94	1.01	2.11	22.39	50.17	62.92	16.00	30.15	0.20	5.76	1.05	2.19	26.94	41.04
69	F	JAN	1100	62.57	55.00	16.25	15.50	0.21	9.20	1.00	2.06	20.93	54.17	62.92	16.00	30.15	0.20	5.76	1.05	2.19	26.94	41.04
70	F	JAN	1100	63.16	55.00	16.00	12.74	0.20	9.46	1.00	2.02	19.47	58.17	62.25	16.58	27.71	0.20	6.49	1.05	2.18	26.17	42.54
71	F	JAN	1100	62.95	48.25	14.75	10.74	0.20	9.54	1.00	2.02	18.29	62.09	62.25	16.58	27.71	0.20	6.49	1.05	2.18	26.17	42.54
72	M	JAN	1100	63.54	41.50	13.50	8.75	0.20	9.63	1.00	2.02	17.11	66.00	61.25	16.58	24.33	0.20	7.26	1.04	2.16	24.74	45.79
79	M	JAN	1200	61.36	81.00	26.50	37.05	0.27	8.38	1.44	2.01	23.83	53.25	82.58	20.08	32.43	0.25	8.82	2.28	2.00	23.15	55.78
80	M	JAN	1200	63.41	78.25	22.75	36.55	0.27	8.26	1.23	2.09	23.57	53.84	82.58	20.08	32.43	0.25	8.82	2.28	2.00	23.15	55.78
82	M	JAN	1200	62.85	69.50	20.25	33.72	0.26	8.43	1.01	2.18	22.87	55.67	81.83	20.17	33.78	0.23	7.65	1.55	2.00	23.64	54.45
84	F	JAN	1200	62.56	63.50	21.50	31.39	0.25	8.72	1.01	2.18	22.44	56.92	79.83	19.00	34.47	0.23	7.44	1.30	2.02	23.87	53.63
87	F	JAN	1200	61.74	47.75	16.00	22.07	0.28	10.17	1.00	2.01	19.10	66.00	78.75	20.83	34.68	0.24	7.79	1.20	2.05	23.83	53.57
88	M	JAN	1200	61.88	39.00	11.50	18.98	0.30	11.10	1.00	2.01	18.26	68.50	74.67	21.25	33.53	0.25	8.29	1.17	2.06	23.17	55.17
89	F	JAN	1200	62.01	41.50	12.25	15.78	0.30	12.25	1.00	1.97	17.67	70.79	74.67	21.25	33.53	0.25	8.29	1.17	2.06	23.17	55.17
90	M	JAN	1200	62.61	44.00	13.00	12.57	0.30	13.39	1.00	1.93	17.08	73.08	67.50	19.75	30.93	0.26	8.90	1.14	2.06	22.08	57.97

101	F	JAN	1100	62.11	85.50	14.50	28.14	0.20	7.97	1.01	2.01	24.47	41.92	81.83	15.83	39.84	0.21	6.71	1.02	2.57	27.91	36.81
103	M	JAN	1100	64.16	74.50	21.50	23.98	0.23	7.98	1.00	2.35	21.66	48.75	82.50	15.42	37.48	0.20	6.71	1.01	2.48	27.41	37.16
104	F	JAN	1100	63.55	74.50	21.50	23.98	0.23	7.98	1.00	2.35	21.66	48.75	82.50	15.42	37.48	0.20	6.71	1.01	2.48	27.41	37.16
105	M	JAN	1100	63.81	41.00	7.00	21.48	0.23	8.82	1.00	2.01	19.70	52.33	82.67	16.25	34.29	0.21	6.96	1.01	2.45	26.34	38.96
106	M	JAN	1100	62.79	41.00	7.00	21.48	0.23	8.82	1.00	2.01	19.70	52.33	82.67	16.25	34.29	0.21	6.96	1.01	2.45	26.34	38.96
107	M	JAN	1100	62.42	44.25	10.00	21.28	0.24	8.77	1.00	1.85	19.11	53.42	82.67	16.25	34.29	0.21	6.96	1.01	2.45	26.34	38.96
108	M	JAN	1100	62.43	47.50	13.00	21.07	0.25	8.73	1.00	1.68	18.52	54.50	76.08	14.75	30.64	0.21	7.31	1.01	2.34	24.85	41.70
109	F	JAN	1100	63.46	43.50	12.75	20.49	0.24	8.35	1.00	1.76	17.96	56.42	76.08	14.75	30.64	0.21	7.31	1.01	2.34	24.85	41.70
118	M	JAN	1200	63.10	49.50	7.00	26.40	0.20	5.77	1.08	2.00	27.20	33.67	55.42	11.50	36.97	0.23	6.52	1.26	2.10	28.56	31.92
121	M	JAN	1100	63.50	44.50	7.00	21.65	0.20	6.02	1.00	2.00	25.74	34.92	54.50	9.92	35.10	0.21	5.53	1.01	2.01	28.63	31.63
122	F	JAN	1100	62.97	41.00	5.50	14.32	0.20	7.10	1.00	1.75	22.63	40.25	53.75	9.00	31.99	0.20	5.43	1.01	2.01	28.23	31.92
123	F	JAN	1100	63.28	37.25	5.50	12.37	0.20	7.18	1.00	1.83	21.31	42.46	53.75	9.00	31.99	0.20	5.43	1.01	2.01	28.23	31.92
124	M	JAN	1100	62.88	33.50	5.50	10.41	0.20	7.27	1.00	1.92	19.98	44.67	51.50	8.00	27.79	0.20	5.64	1.01	1.97	27.14	33.40
125	M	JAN	1100	63.71	33.50	5.50	10.41	0.20	7.27	1.00	1.92	19.98	44.67	51.50	8.00	27.79	0.20	5.64	1.01	1.97	27.14	33.40
126	F	JAN	1100	62.39	39.25	7.25	9.78	0.20	7.48	1.00	1.96	19.22	47.50	51.50	8.00	27.79	0.20	5.64	1.01	1.97	27.14	33.40
127	M	JAN	1100	62.93	45.00	9.00	9.16	0.20	7.69	1.00	2.00	18.46	50.33	47.92	7.67	23.62	0.20	6.02	1.01	1.96	25.57	35.85
137	M	FEB	1200	62.96	117.00	19.50	35.89	0.20	6.31	1.00	2.00	26.93	25.88	91.17	16.17	46.74	0.22	7.59	1.21	2.31	30.28	20.71
138	F	FEB	1100	63.09	104.50	19.50	34.39	0.20	6.85	1.00	2.00	26.18	26.67	98.00	15.58	44.44	0.20	6.16	1.00	2.15	29.99	21.21
139	M	FEB	1100	63.49	104.50	19.50	34.39	0.20	6.85	1.00	2.00	26.18	26.67	98.00	15.58	44.44	0.20	6.16	1.00	2.15	29.99	21.21
140	M	FEB	1100	61.04	149.50	39.50	33.72	0.29	8.66	1.00	2.00	22.92	35.83	102.00	15.50	41.08	0.20	6.05	1.00	2.14	29.24	22.03
141	M	FEB	1100	61.99	149.50	39.50	33.72	0.29	8.66	1.00	2.00	22.92	35.83	102.00	15.50	41.08	0.20	6.05	1.00	2.14	29.24	22.03
142	M	FEB	1200	62.29	109.50	35.50	29.48	0.34	10.89	1.00	2.00	20.79	41.42	114.17	20.17	38.12	0.22	6.38	1.00	2.01	27.93	24.45
143	F	FEB	1100	61.87	109.50	35.50	29.48	0.34	10.89	1.00	2.00	20.79	41.42	114.17	20.17	38.12	0.22	6.38	1.00	2.01	27.93	24.45
144	M	FEB	1100	61.42	104.00	41.00	24.48	0.40	12.38	1.00	2.00	19.48	44.25	119.33	24.42	35.88	0.24	7.23	1.00	2.01	26.25	28.15
145	F	FEB	1200	62.65	104.00	41.00	24.48	0.40	12.38	1.00	2.00	19.48	44.25	119.33	24.42	35.88	0.24	7.23	1.00	2.01	26.25	28.15
155	M	FEB	1100	62.14	107.50	1.00	39.76	0.23	8.98	1.00	3.44	30.93	18.13	83.75	22.06	55.69	0.29	10.17	1.35	2.97	32.93	18.96
156	F	FEB	1100	61.22	134.50	2.00	39.22	0.21	9.35	1.00	2.98	29.77	20.25	81.67	17.06	52.17	0.27	7.73	1.00	2.99	33.03	17.94
157	F	FEB	1100	63.73	125.00	16.75	35.89	0.23	10.46	1.00	3.19	28.26	22.75	81.67	17.06	52.17	0.27	7.73	1.00	2.99	33.03	17.94
158	M	FEB	1100	61.18	115.50	31.50	32.56	0.25	11.58	1.00	3.39	26.75	25.25	92.58	13.47	48.41	0.26	7.91	1.00	3.04	32.49	18.11
160	M	FEB	1100	61.26	143.00	37.00	26.65	0.36	16.30	1.00	2.98	24.47	32.75	98.75	14.31	43.49	0.25	8.69	1.00	3.16	31.38	19.19
161	M	FEB	1100	62.21	143.00	37.00	26.65	0.36	16.30	1.00	2.98	24.47	32.75	98.75	14.31	43.49	0.25	8.69	1.00	3.16	31.38	19.19
162	M	FEB	1100	60.88	82.50	32.50	19.65	0.38	20.11	1.00	2.90	22.99	35.25	108.17	16.39	38.93	0.26	10.12	1.00	3.23	29.86	21.49
163	M	FEB	1100	60.95	82.50	32.50	19.65	0.38	20.11	1.00	2.90	22.99	35.25	108.17	16.39	38.93	0.26	10.12	1.00	3.23	29.86	21.49
168	M	FEB	1100	62.97	69.50	18.25	39.18	0.19	4.92	1.00	2.01	29.86	38.79	72.63	19.38	35.64	0.21	9.14	2.58	2.40	26.94	43.34
173	M	FEB	1000	63.62	66.00	21.00	28.57	0.19	5.14	1.00	2.01	27.20	45.31	70.90	18.40	39.64	0.19	6.00	1.03	2.14	28.88	39.85
188	F	FEB	1200	62.59	64.50	29.50	34.31	0.20	6.28	1.42	1.58	28.56	40.08	56.67	21.08	35.67	0.24	10.02	3.54	1.44	27.80	42.15
191	M	FEB	1000	62.60	61.00	14.00	30.23	0.20	7.12	1.42	1.58	27.52	41.75	58.25	21.17	37.33	0.21	7.45	1.80	1.40	28.44	40.69
193	M	FEB	1000	62.98	47.50	18.50	23.31	0.20	6.95	1.09	2.00	25.70	44.67	59.92	17.75	36.67	0.20	6.84	1.46	1.40	28.56	40.40
194	F	FEB	1000	62.86	48.25	15.50	17.15	0.20	8.29	1.05	1.71	23.84	49.00	59.92	17.75	36.67	0.20	6.84	1.46	1.40	28.56	40.40
195	F	FEB	1000	61.89	49.00	12.50	10.99	0.20	9.63	1.00	1.42	21.97	53.33	56.00	17.92	34.03	0.20	6.45	1.26	1.53	28.15	40.83

196	M	FEB	1000	63.21	31.25	8.75	9.41	0.17	8.71	1.00	1.29	20.97	54.00	56.00	17.92	34.03	0.20	6.45	1.26	1.53	28.15	40.83
197	M	FEB	1000	62.38	13.50	5.00	7.83	0.14	7.79	1.00	1.17	19.97	54.67	55.75	17.83	28.99	0.20	6.98	1.24	1.60	26.98	43.06
198	F	FEB	1000	63.06	22.75	6.50	6.70	0.16	7.32	1.09	1.08	19.54	55.80	55.75	17.83	28.99	0.20	6.98	1.24	1.60	26.98	43.06
199	M	FEB	1200	62.45	32.00	8.00	5.58	0.17	6.86	1.17	1.00	19.10	56.92	48.92	16.33	23.68	0.19	7.25	1.24	1.57	25.47	45.65
200	F	FEB	1200	63.15	32.00	8.00	5.58	0.17	6.86	1.17	1.00	19.10	56.92	48.92	16.33	23.68	0.19	7.25	1.24	1.57	25.47	45.65
212	F	FEB	1000	62.60	82.00	20.00	25.19	0.32	10.21	1.00	2.01	20.91	57.96	88.67	19.33	35.22	0.31	8.10	1.22	2.02	24.45	49.45
213	F	FEB	1000	64.00	72.00	17.50	22.23	0.31	9.58	1.00	2.01	20.34	58.50	91.25	19.67	34.32	0.31	8.35	1.00	2.01	23.99	50.49
214	M	FEB	1000	61.96	60.25	17.25	20.86	0.31	9.83	1.00	1.93	19.82	59.38	91.25	19.67	34.32	0.31	8.35	1.00	2.01	23.99	50.49
215	M	FEB	1200	62.55	48.50	17.00	19.48	0.30	10.08	1.00	1.84	19.30	60.25	93.58	20.00	31.65	0.31	8.66	1.00	2.01	23.20	52.29
216	M	FEB	1000	62.84	48.50	17.00	19.48	0.30	10.08	1.00	1.84	19.30	60.25	93.58	20.00	31.65	0.31	8.66	1.00	2.01	23.20	52.29
217	F	FEB	1000	62.37	50.00	17.00	10.41	0.35	19.25	1.00	1.59	17.81	66.58	86.50	19.92	28.93	0.31	8.90	1.00	1.98	22.24	54.45
218	M	FEB	1200	62.00	50.00	17.00	10.41	0.35	19.25	1.00	1.59	17.81	66.58	86.50	19.92	28.93	0.31	8.90	1.00	1.98	22.24	54.45
246	M	MAY	1200	62.99	164.25	40.00	21.19	0.61	50.43	1.79	4.05	20.41	49.21	124.25	45.92	39.32	0.92	76.06	20.85	5.77	26.04	23.64
247	M	MAY	1200	62.43	164.50	38.00	13.99	0.59	51.39	1.83	3.97	18.86	53.83	127.92	44.00	41.54	0.83	69.93	13.22	5.34	25.92	25.67
249	M	MAY	1000	61.92	232.00	45.00	1.83	0.97	72.50	21.25	3.97	17.80	56.33	134.67	41.67	40.11	0.74	62.68	7.42	4.98	24.88	30.33
250	M	MAY	1000	61.29	232.00	45.00	1.83	0.97	72.50	21.25	3.97	17.80	56.33	134.67	41.67	40.11	0.74	62.68	7.42	4.98	24.88	30.33
251	M	MAY	1200	61.56	272.00	51.00	1.00	1.17	67.05	23.08	3.97	15.53	64.00	152.33	40.17	34.90	0.71	57.71	6.74	4.65	23.35	36.17
252	F	MAY	1000	61.59	272.00	51.00	1.00	1.17	67.05	23.08	3.97	15.53	64.00	152.33	40.17	34.90	0.71	57.71	6.74	4.65	23.35	36.17
253	F	MAY	1000	61.07	234.75	55.75	1.62	1.87	71.12	82.29	5.04	14.88	66.79	152.33	40.17	34.90	0.71	57.71	6.74	4.65	23.35	36.17
254	M	MAY	1000	61.30	197.50	60.50	2.25	2.57	75.18	141.50	6.11	14.22	69.58	183.08	42.83	26.77	0.77	57.49	8.94	4.41	21.22	43.90
255	F	MAY	1000	60.95	202.25	63.25	2.12	2.62	75.62	167.25	6.48	13.70	71.75	183.08	42.83	26.77	0.77	57.49	8.94	4.41	21.22	43.90
265	F	MAY	1100	62.17	69.50	26.00	3.50	0.66	46.75	13.85	1.17	16.78	66.71	75.83	27.33	11.98	0.71	41.55	19.77	3.88	18.72	55.22
301	M	MAY	1000	61.94	111.50	23.50	8.58	0.36	36.35	5.45	2.91	17.60	45.67	86.50	27.08	15.54	0.55	46.46	33.30	3.28	20.07	38.20
302	F	MAY	1200	62.79	111.00	24.50	5.33	0.41	37.10	7.77	2.91	16.07	51.88	86.50	27.08	15.54	0.55	46.46	33.30	3.28	20.07	38.20
303	M	MAY	1200	61.90	110.50	25.50	2.08	0.45	37.85	10.08	2.91	14.54	58.08	83.83	23.00	15.85	0.43	41.75	18.64	3.10	20.35	36.72
305	M	MAY	1000	62.24	168.50	28.00	2.00	0.62	35.98	23.46	2.91	13.00	60.42	87.83	21.42	14.35	0.38	37.66	11.52	3.03	19.63	39.20
306	M	MAY	1000	60.93	168.50	28.00	2.00	0.62	35.98	23.46	2.91	13.00	60.42	87.83	21.42	14.35	0.38	37.66	11.52	3.03	19.63	39.20
307	F	MAY	1000	61.20	145.25	29.50	2.00	0.73	36.97	39.94	3.08	12.47	61.21	87.83	21.42	14.35	0.38	37.66	11.52	3.03	19.63	39.20
308	F	MAY	1000	60.93	122.00	31.00	2.00	0.85	37.96	56.42	3.24	11.93	62.00	108.83	23.92	11.60	0.41	37.31	12.01	3.05	18.39	43.43
309	F	MAY	1000	61.30	109.50	33.75	2.00	1.06	37.57	84.46	3.83	11.43	62.21	108.83	23.92	11.60	0.41	37.31	12.01	3.05	18.39	43.43
310	F	MAY	1200	61.79	97.00	36.50	2.00	1.27	37.17	112.50	4.41	10.93	62.42	120.83	27.17	8.60	0.51	38.58	19.18	3.08	16.73	48.85
318	M	MAY	1100	63.32	130.25	36.00	39.80	0.47	25.33	1.00	3.00	18.98	33.88	67.92	30.08	35.54	0.57	34.33	9.79	3.31	20.20	29.21
322	M	MAY	1000	62.82	126.50	36.00	21.40	0.77	34.08	1.42	2.92	16.44	45.25	84.33	29.67	42.56	0.50	30.43	3.86	3.22	20.96	27.74
324	M	MAY	1100	62.26	127.00	40.00	14.90	0.69	35.67	1.00	2.25	15.41	47.50	94.92	30.67	40.63	0.52	30.03	2.69	3.14	20.27	30.39
325	F	MAY	1100	62.02	91.50	30.50	10.03	0.65	34.42	1.42	2.67	15.08	46.54	94.92	30.67	40.63	0.52	30.03	2.69	3.14	20.27	30.39
326	M	MAY	1100	60.34	56.00	21.00	5.16	0.61	33.17	1.83	3.08	14.74	45.58	105.42	32.17	36.72	0.54	28.85	1.51	2.93	19.10	34.25
327	F	MAY	1100	60.12	51.00	21.00	3.62	0.57	31.46	2.83	3.04	13.76	49.25	105.42	32.17	36.72	0.54	28.85	1.51	2.93	19.10	34.25
328	M	MAY	1100	60.48	46.00	21.00	2.08	0.52	29.75	3.83	3.00	12.77	52.92	105.25	32.00	28.99	0.56	29.31	1.25	2.88	17.70	38.35
452	M	JUL	1100	61.48	130.50	37.50	2.75	0.80	48.75	19.12	2.88	17.12	46.83	125.75	48.92	8.20	1.10	79.65	70.09	2.50	19.86	34.39
453	F	JUL	1100	61.16	130.50	37.50	2.75	0.80	48.75	19.12	2.88	17.12	46.83	125.75	48.92	8.20	1.10	79.65	70.09	2.50	19.86	34.39

454	F	JUL	1100	63.06	90.50	24.00	1.00	0.75	38.00	14.63	2.96	13.99	56.33	113.25	40.75	8.12	0.89	70.28	42.07	2.26	19.93	33.86
455	M	JUL	1100	62.04	90.50	24.00	1.00	0.75	38.00	14.63	2.96	13.99	56.33	113.25	40.75	8.12	0.89	70.28	42.07	2.26	19.93	33.86
456	F	JUL	1100	61.88	98.00	31.00	1.17	0.76	37.42	18.38	2.88	12.56	56.75	107.00	35.75	7.23	0.81	62.17	29.79	2.27	19.07	36.43
457	M	JUL	1100	62.46	98.00	31.00	1.17	0.76	37.42	18.38	2.88	12.56	56.75	107.00	35.75	7.23	0.81	62.17	29.79	2.27	19.07	36.43
458	M	JUL	1100	61.24	135.00	36.25	1.33	0.94	37.96	40.83	3.17	11.95	58.59	107.00	35.75	7.23	0.81	62.17	29.79	2.27	19.07	36.43
459	F	JUL	1100	62.05	172.00	41.50	1.50	1.13	38.50	63.28	3.47	11.33	60.42	108.67	34.08	5.77	0.77	54.90	24.33	2.38	17.66	40.65
460	F	JUL	1100	62.43	162.25	45.00	1.75	1.31	39.00	85.29	3.77	10.49	63.71	108.67	34.08	5.77	0.77	54.90	24.33	2.38	17.66	40.65
461	M	JUL	1100	62.12	152.50	48.50	2.00	1.50	39.50	107.30	4.06	9.64	67.00	121.33	34.67	4.07	0.81	48.36	29.23	2.72	16.05	46.51
462	M	JUL	1100	61.33	134.50	49.50	2.04	1.55	39.00	110.80	4.06	9.38	68.00	121.33	34.67	4.07	0.81	48.36	29.23	2.72	16.05	46.51
468	M	JUL	1200	63.05	119.00	38.50	16.26	0.57	60.96	16.27	6.50	19.56	30.13	158.17	65.67	10.70	1.49	76.96	#####	4.85	13.89	47.82
471	M	JUL	1100	63.93	142.50	40.50	6.62	0.49	50.08	9.99	4.42	16.46	41.17	133.50	51.25	15.13	0.89	70.79	45.91	4.74	17.43	36.99
473	M	JUL	1100	61.74	125.00	31.50	1.90	0.61	48.92	28.83	4.00	13.17	48.08	130.00	46.83	15.20	0.74	65.86	30.09	4.75	18.02	35.46
474	M	JUL	1100	61.56	125.00	31.50	1.90	0.61	48.92	28.83	4.00	13.17	48.08	130.00	46.83	15.20	0.74	65.86	30.09	4.75	18.02	35.46
475	F	JUL	1100	62.09	96.25	22.75	1.95	0.58	44.09	28.67	3.67	12.24	44.54	130.00	46.83	15.20	0.74	65.86	30.09	4.75	18.02	35.46
476	M	JUL	1200	62.14	67.50	14.00	1.99	0.56	39.25	28.50	3.33	11.30	41.00	123.75	40.42	14.09	0.62	58.86	20.43	4.75	17.58	36.47
477	M	JUL	1100	62.76	79.25	16.00	1.95	0.54	36.04	28.05	3.17	10.11	42.50	123.75	40.42	14.09	0.62	58.86	20.43	4.75	17.58	36.47
478	M	JUL	1100	62.67	91.00	18.00	1.90	0.52	32.83	27.59	3.00	8.92	44.00	113.17	33.75	11.14	0.57	53.24	19.55	4.78	16.50	37.18
479	M	JUL	1100	62.61	87.00	19.25	1.95	0.55	31.92	32.38	2.88	7.73	49.13	113.17	33.75	11.14	0.57	53.24	19.55	4.78	16.50	37.18
480	M	JUL	1100	62.95	83.00	20.50	1.99	0.58	31.00	37.17	2.75	6.54	54.25	110.67	30.17	7.49	0.55	48.83	21.24	4.63	14.83	39.08
481	M	JUL	1100	63.36	83.00	20.50	1.99	0.58	31.00	37.17	2.75	6.54	54.25	110.67	30.17	7.49	0.55	48.83	21.24	4.63	14.83	39.08
492	F	JUL	1100	64.13	112.50	30.50	6.04	0.22	42.00	3.84	2.00	13.51	47.25	98.58	28.42	17.67	0.36	43.72	17.50	3.21	18.14	29.14
493	M	JUL	1100	62.13	112.50	30.50	6.04	0.22	42.00	3.84	2.00	13.51	47.25	98.58	28.42	17.67	0.36	43.72	17.50	3.21	18.14	29.14
494	F	JUL	1100	62.55	102.00	31.00	4.26	0.26	43.71	5.85	2.04	12.89	49.67	98.58	28.42	17.67	0.36	43.72	17.50	3.21	18.14	29.14
495	F	JUL	1100	62.31	91.50	31.50	2.48	0.30	45.42	7.85	2.08	12.27	52.08	100.00	27.83	16.88	0.25	40.04	8.95	2.87	17.39	32.35
496	M	JUL	1100	62.70	78.50	30.75	4.18	0.29	41.38	5.60	1.95	11.68	55.04	100.00	27.83	16.88	0.25	40.04	8.95	2.87	17.39	32.35
497	M	JUL	1100	62.62	65.50	30.00	5.88	0.28	37.33	3.34	1.83	11.08	58.00	101.92	28.50	14.65	0.20	38.00	5.57	2.65	16.18	37.29
498	F	JUL	1100	63.01	56.75	28.75	6.41	0.24	33.79	2.34	1.71	10.32	61.09	101.92	28.50	14.65	0.20	38.00	5.57	2.65	16.18	37.29
499	F	JUL	1100	62.61	48.00	27.50	6.95	0.19	30.25	1.34	1.58	9.55	64.17	100.92	30.58	11.29	0.22	38.47	4.72	2.47	14.72	43.61
500	M	JUL	1100	63.28	48.00	27.50	6.95	0.19	30.25	1.34	1.58	9.55	64.17	100.92	30.58	11.29	0.22	38.47	4.72	2.47	14.72	43.61
530	F	JUN	1100	60.76	74.50	15.50	2.00	0.47	29.67	33.42	2.35	8.54	50.08	49.08	8.75	11.77	0.34	29.94	16.08	2.52	12.82	45.57
531	M	JUN	1100	62.88	55.00	15.50	2.00	0.50	26.83	30.92	1.59	7.53	52.75	54.75	8.92	9.93	0.36	30.57	18.01	2.47	12.36	45.10
532	F	JUN	1100	61.27	55.00	15.50	2.00	0.50	26.83	30.92	1.59	7.53	52.75	54.75	8.92	9.93	0.36	30.57	18.01	2.47	12.36	45.10
533	M	JUN	1100	61.19	67.50	19.50	2.00	0.84	28.33	62.58	1.59	5.78	60.83	55.92	11.50	8.04	0.39	30.40	19.78	2.30	11.46	45.93
534	F	JUN	1100	62.47	67.50	19.50	2.00	0.84	28.33	62.58	1.59	5.78	60.83	55.92	11.50	8.04	0.39	30.40	19.78	2.30	11.46	45.93
535	F	JUN	1100	61.92	74.50	26.25	2.21	0.98	28.92	80.08	2.06	5.47	63.92	55.92	11.50	8.04	0.39	30.40	19.78	2.30	11.46	45.93
536	F	JUN	1100	62.17	81.50	33.00	2.42	1.13	29.50	97.58	2.52	5.17	67.00	58.58	14.33	5.95	0.48	30.31	27.29	2.18	10.13	48.82
558	F	AUG	1100	63.82	25.50	5.50	18.12	0.20	13.50	2.83	1.00	9.19	66.67	44.08	14.00	12.41	0.53	21.26	37.80	1.86	8.19	72.51
564	M	AUG	1200	62.74	39.25	8.25	4.59	0.36	26.55	8.85	1.04	7.59	72.67	22.00	4.67	16.55	0.23	13.97	4.61	1.03	8.77	69.75
566	M	AUG	1200	62.10	41.50	9.00	1.90	0.42	29.42	13.05	1.08	7.17	73.83	23.92	4.92	15.53	0.24	15.36	4.39	1.00	8.82	69.20
568	M	AUG	1100	62.13	63.50	14.50	1.99	0.62	29.75	35.17	1.66	6.60	77.42	27.75	5.92	13.14	0.27	18.15	5.61	1.01	8.65	69.21

569	M	AUG	1100	61.48	*81.25*	*18.25*	*1.99*	*0.73*	*28.42*	*49.31*	*1.91*	*6.06*	*80.00*	**27.75**	**5.92**	**13.14**	**0.27**	**18.15**	**5.61**	**1.01**	**8.65**	**69.21**
570	M	AUG	1100	62.86	*99.00*	*22.00*	*1.99*	*0.85*	*27.08*	*63.44*	*2.16*	*5.52*	*82.58*	**35.33**	**7.58**	**10.52**	**0.33**	**20.92**	**10.63**	**1.12**	**8.21**	**70.72**
572	M	AUG	1100	62.18	*121.00*	*26.00*	*1.99*	*1.23*	*31.33*	*117.60*	*3.24*	*5.50*	*82.92*	**48.33**	**10.50**	**7.85**	**0.44**	**23.13**	**20.44**	**1.32**	**7.53**	**73.50**
573	M	AUG	1100	62.74	*99.50*	*27.25*	*1.99*	*1.49*	*32.54*	*139.55*	*3.65*	*5.34*	*82.88*	**48.33**	**10.50**	**7.85**	**0.44**	**23.13**	**20.44**	**1.32**	**7.53**	**73.50**

Italic style corresponds to the values at the time of the races, while in bold are the values for the six hours before them.
